# Association of FKBP5 gene methylation and adolescents’ sex with depressive symptoms outcomes: a nested case-control study among Chinese adolescents

**DOI:** 10.1192/j.eurpsy.2022.395

**Published:** 2022-09-01

**Authors:** W. Li, C. Lu

**Affiliations:** Sun Yat-Sen university, Department Of Medical Statistics And Epidemiology, School Of Public Health, Guangzhou, China

**Keywords:** FKBP5, DNA methylation, depressive symptoms, sex differences

## Abstract

**Introduction:**

Altered DNA methylation in the FK506 binding protein 5 (*FKBP5*) gene has been shown to regulate stress response, which may serve as a biomarker of depression and a promising candidate for explaining sexual differences. However, there is no consistent conclusion so far.

**Objectives:**

The present study aimed to test the associations of *FKBP5* DNA methylation with depressive symptoms and whether these associations were influenced by sex.

**Methods:**

A nested case-control study comprising 87 cases and 151 controls was conducted in South China from January 2019 and December 2019. Peripheral blood for DNA extraction and DNA methylation analysis of *FKBP5* gene promoter was collected, and severity of depressive symptoms was assessed at baseline and after one year follow-up.

**Results:**

Compared to healthy controls, lower methylation percentage of *FKBP5*-12 CpG 1 was observed in adolescents with depressive symptoms after adjusting covariates (case: 0.94 ± 2.00, control: 0.47 ± 0.92; *F* = 5.41, *P* = 0.021). In addition, hypomethylation of *FKBP5* CpG sites was not an independent risk factor for depressive symptoms after adjustment for environmental stress factors (*P* > 0.05). No significant sex differences were found in the association of *FKBP5* gene methylation with depressive symptoms.

**Conclusions:**

Lower levels of *FKBP5* methylation were found in adolescents with depressive symptoms. Our study supported that the epigenetic factors did not act alone in the development of depressive symptoms. Taken together, these findings contribute to a better understanding of complex mechanisms of gene-environment interactions involved in depression.

**Disclosure:**

No significant relationships.

